# Cell-Type-Specific Expression of Leptin Receptors in the Mouse Forebrain

**DOI:** 10.3390/ijms25189854

**Published:** 2024-09-12

**Authors:** Cade R. Canepa, John A. Kara, Charles C. Lee

**Affiliations:** Department of Comparative Biomedical Sciences, School of Veterinary Medicine, Louisiana State University, Baton Rouge, LA 70803, USA

**Keywords:** leptin, auditory, somatosensory, visual, hippocampus, claustrum

## Abstract

Leptin is a hormone produced by the small intestines and adipose tissue that promotes feelings of satiety. Leptin receptors (LepRs) are highly expressed in the hypothalamus, enabling central neural control of hunger. Interestingly, LepRs are also expressed in several other regions of the body and brain, notably in the cerebral cortex and hippocampus. These brain regions mediate higher-order sensory, motor, cognitive, and memory functions, which can be profoundly altered during periods of hunger and satiety. However, LepR expression in these regions has not been fully characterized on a cell-type-specific basis, which is necessary to begin assessing their potential functional impact. Consequently, we examined LepR expression on neurons and glia in the forebrain using a LepR-Cre transgenic mouse model. LepR-positive cells were identified using a ‘floxed’ viral cell-filling approach and co-labeling immunohistochemically for cell-type-specific markers, i.e., NeuN, VGlut2, GAD67, parvalbumin, somatostatin, 5-HT3R, WFA, S100β, and GFAP. In the cortex, LepR-positive cells were localized to lower layers (primarily layer 6) and exhibited non-pyramidal cellular morphologies. The majority of cortical LepR-positive cells were neurons, while the remainder were identified primarily as astrocytes or other glial cells. The majority of cortical LepR-positive neurons co-expressed parvalbumin, while none expressed somatostatin or 5-HT3R. In contrast, all hippocampal LepR-positive cells were neuronal, with none co-expressing GFAP. These data suggest that leptin can potentially influence neural processing in forebrain regions associated with sensation and limbic-related functions.

## 1. Introduction

Leptin (Lep) promotes satiety through multiple physiological mechanisms, while its dysfunctional regulation is associated with obesity [1]. Leptin is produced in adipose tissue, enters circulation, and binds to cellular leptin receptors (LepRs) [2]. Multiple LepR isoforms can be expressed, generally ‘short’ and ‘long’, dependent on alternative splicing events [3]. Functionally, the ‘short’ isoform assists with the transport of Lep across the blood–brain barrier [4,5], while the ‘long’ isoform (LepRb) is responsible for the majority of leptin’s physiological effects [6,7]. The various cell-types expressing LepRs mediate its importance in physiological homeostasis.

In the brain, LepRb is highly expressed in the basomedial hypothalamus, notably the arcuate nucleus (ARC), as part of the brain’s feeding circuitry [8,9]. LepRb in the ARC can trigger two different effects, depending on neuronal cell-type [10]. First, neurons that co-express neuropeptide Y (NPY) and agouti-related peptide (AgRP) exert inhibitory effects via STAT3 inhibition of AgRP production, which in turn disinhibits proopiomelanocortin (POMC). Second, in neurons that lack NPY and AgRP, POMC expression is stimulated to promote satiety and appetite-suppression [10].

Although LepRb’s role in the ARC is well studied, the receptor is also expressed elsewhere throughout the brain and the body [11], with these “extrahypothalmic LepRs” receiving less attention. In particular, LepRs are present in the forebrain, notably in the cerebral cortex and hippocampus, which are involved in the higher processing of sensory information, learning, memory, and other cognitive functions. LepR expression in the forebrain can potentially influence several neural functions involved in feeding-related behaviors.

In particular, the cerebral cortex, the outermost portion of the brain, is responsible for many higher sensory and cognitive functions [12]. The laminar organization throughout the neocortex can be organized into six main layers based on the cellular composition and connectional architecture [13]. Notably, the lower cortical layers, layers 5 and 6, are the source for descending pathways to the brainstem, thalamus, and other subcortical structures [12,14]. On a larger scale, the cerebral cortex can also be divided into functionally specific areas. For instance, the somatosensory, auditory, and visual cortices are responsible for touch, hearing, and vision, respectively [15,16,17,18].

Leptin and its receptor are highly conserved across mammalian species, with similar relevance to human physiology [19,20,21,22]. Thus, to assess the cell-type-specific expression of LepRs, we employed a Cre-transgenic mouse model to identify LepR-positive cells in the forebrain. LepR-positive cells were characterized morphologically and double-labeled for neuronal and glial markers to identify specific cell-types. Overall, our studies highlight a unique organization of LepR-expressing cells in the sensory and limbic forebrain regions of the mouse.

## 2. Results

### 2.1. Cerebral Cortex

**Figure 1:**
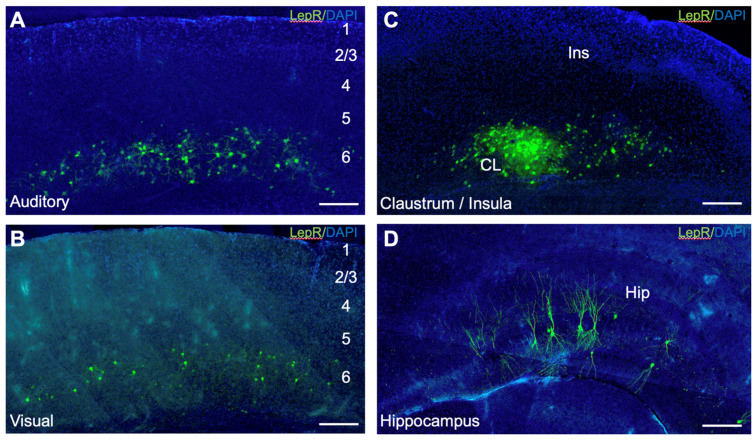
Leptin receptor (LepR)-positive cells in the mouse forebrain. (**A**) Primary auditory cortex; (**B**) primary visual cortex; (**C**) insular cortex and claustrum; (**D**) hippocampus. Green labeling: LepR-positive cells. Blue labeling: DAPI counterstain. Scale bar: 200 μm.

To identify LepR-positive cortical cells, we injected a ‘floxed’ AAV vector expressing a fluorophore in LepR-Cre transgenic mice (see Methods, Figure 1, Supplementary Figure S1).

**Figure 2:**
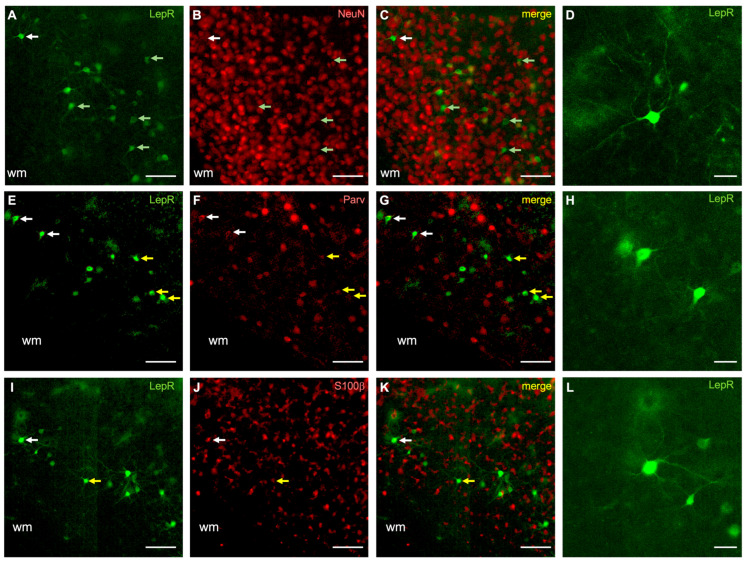
LepR-positive cells in sensory cortices co-localize with NeuN, parvalbumin, and S100β. (**A**–**D**) Neuronal marker (NeuN) co-staining; (**E**–**H**) parvalbumin co-staining; (**I**–**L**) S100β co-staining. In all panels, the green rendering indicates LepR-positive cells (**A**,**E**,**I**), the red rendering indicates neurochemical markers (**B**,**F**,**J**), and the yellow rendering illustrates double-labeling (**C**,**G**,**K**). The dendritic morphology of representative cells (**D**,**H**,**L**) is indicated by the white arrows in their respective rows. Light green arrows indicate non-double-labeled cells (**A**–**C**). Yellow arrows indicate double-labeled cells (**E**–**G**,**I**–**K**). Scale bar in (**G**,**H**,**L**): 10 μm. Scale bar in other panels: 100 μm. wm: white matter.

Following viral transfection, LepR-positive cells were found in the lower cortical layers of all areas injected (Figure 1A–C; Supplementary Figure S1). The labeled cells were primarily concentrated in layer 6, spanning the areal extent of the injection. Many LepR-positive cells were found near the white matter, presumably localized to layer 6b (Figure 1A–C; Supplementary Figure S1). No labeled cells were found in upper cortical layers 1–4 (Figure 1). Morphologically, LepR-positive cells exhibited radial or stellate arborizations that were focally organized within laminae, extending ~100 μm from the soma with critical values of ~50 μm (Figure 2D,H,L; Supplementary Figure S5A–C). These cells lacked obvious long-range fiber projections, suggesting roles confined to local cortical operations (Figure 2D,H,L).

**Figure 3:**
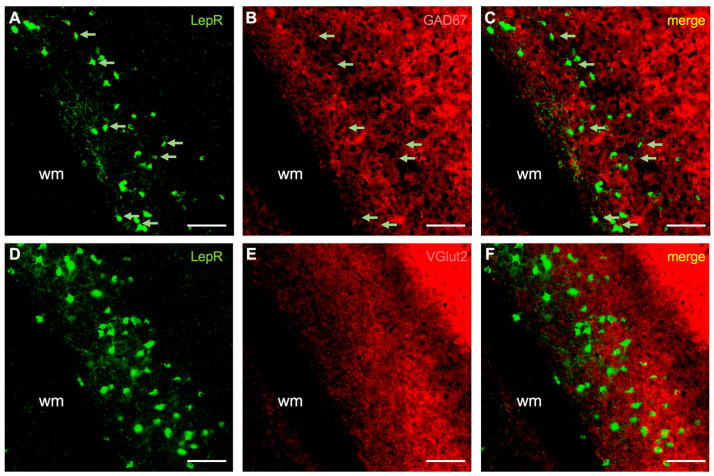
Co-labeling of LepR-positive cells with GAD67 and VGlut2. (**A**–**C**) Marker for GABAergic inhibitory neurons (GAD67). (**D**–**F**) VGlut2 co-staining. The green rendering indicates LepR-positive cells (**A**,**C**,**D**,**F**), the red rendering indicates neurochemical markers (**B**,**C**,**E**,**F**), and the yellow rendering indicates double-labeling (**C**,**F**). Light green arrows indicate non-double-labeled cells (**A**–**C**). Scale bar: 100 μm. wm: white matter.

**Figure 4:**
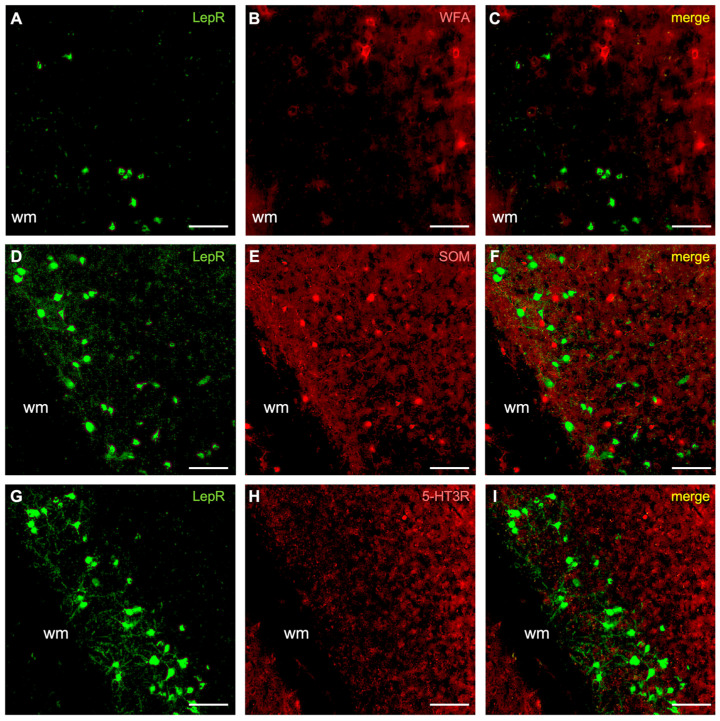
LepR-positive cells co-stained for perineuronal nets (WFA), somatostatin (SOM) and serotonin receptor 3R (5-HT3R). (**A**–**C**) Perineuronal net (WFA) co-staining. (**D**–**F**) Somatostatin (SOM), calcium binding protein, co-staining. (**G**–**I**) Co-staining for somatostatin (SOM) calcium binding protein. The green rendering depicts LepR-positive cells (**A**,**D**,**G**), the red rendering is for the neurochemical markers (**B**,**E**,**H**), and the yellow rendering illustrates double-labeling (**C**,**F**,**I**). Scale bar: 100 μm. wm: white matter.

We then investigated the cell phenotype of LepR-positive cells by immunostaining for NeuN, S100β, GAD67, VGlut2, parvalbumin, somatostatin, 5-HT3R, and WFA (Figure 2, Figure 3 and Figure 4; Supplementary Figures S2–S4). Co-localization was quantified as the percentage of LepR-positive cells that were double-labeled with these respective markers (see Methods). Of these markers, LepR-positive cells colocalized with the neuronal marker (NeuN: 76.6 ± 11.1%), the astrocytic marker (S100β: 16.6 ± 4.0%), and the marker for GABAergic inhibitory neurons (GAD67: 80.3 ± 16.1%) (Figure 2 and Figure 3; Supplementary Figures S2, S3 and S5E). Immunostaining for VGlut2 resulted in a dense band of puncta labeled in cortical layers 4 and 6, which was found to be juxtaposed with most LepR-positive cells (Figure 3E–G, Supplementary Figure S3B).

Our results suggest that most LepR-positive cortical cells are inhibitory interneurons (~80%). We therefore sought to further classify these based on their co-labeling for three major inhibitory neuronal subtype markers: parvalbumin, somatostatin, and the serotonin type 3 receptor (5-HT3R). First, we found that many LepR-positive cells were co-labeled for parvalbumin (32.2 ± 12.4%) (Figure 2E–H, Supplementary Figure S2B). Due to their high metabolic demand, parvalbumin interneurons are often surrounded by perineuronal nets (PNNs) [23,24]. Therefore, we co-stained for PNNs; however, these were not observed to surround LepR-positive cells (Figure 4A–C, Supplementary Figure S4A). Furthermore, we did not observe co-labeling with either somatostatin (Figure 4D–F, Supplementary Figure S4B) or 5-HT3R (Figure 4G–I, Supplementary Figure S4C). These data identify a significant fraction of LepR-positive cortical cells as parvalbumin-positive interneurons (~30%), although the remaining identity of LepR-positive interneuronal subtypes is unresolved.

### 2.2. Hippocampus

**Figure 5:**
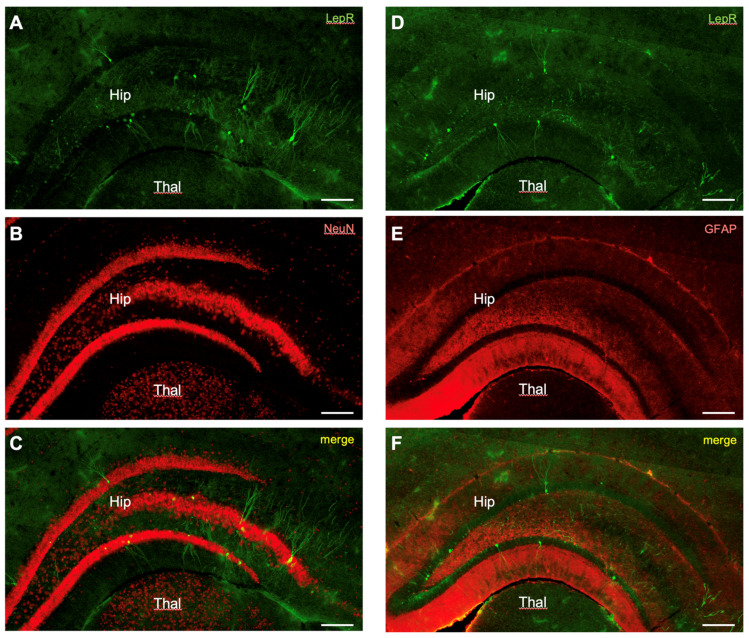
LepR-positive cells in the hippocampus co-stained for NeuN and GFAP. (**A**–**C**) Co-labeling of LepR-positive cells with NeuN. (**D**–**F**) Co-labeling of LepR-positive cells for GFAP. The green rendering depicts LepR-positive cells (**A**,**D**), the red rendering is for the neurochemical marker (**B**,**E**), and the yellow rendering illustrates double-labeling (**C**,**F**). Scale bar: 100 μm. Hip: hippocampus. Thal: thalamus.

We also broadly surveyed the LepR-positive cells in the hippocampal CA3 subfield, the granular cell layer of the dentate gyrus (GrDG), and the polymorphic layer of the dentate gyrus (PoDG). LepR-positive cells were found throughout these regions and were not localized to any particular subregion. Morphologically, the LepR-positive cells had long, branching projections from the soma, which extended ~300 µm from the soma, with critical values of ~150 μm (Supplementary Figure S5D). These projections were orthogonally polarized relative to the hippocampal laminae (Figure 5, Supplementary Figure S1E).

We broadly surveyed the cell phenotype of the labeled cells by co-localizing LepR-expressing cells with immunostaining for neurons (NeuN) and astrocytes (GFAP). Cell types were far more uniform in the hippocampus with 90.7 ± 6.6% of LepR-positive cells co-labeled with NeuN (Figure 5A–C). None of the LepR-positive cells in the hippocampus were found to co-localize with GFAP (Figure 5D–F).

## 3. Discussion

In this study, we investigated the distribution and neurochemical identity of LepR-positive cells in the mouse forebrain. We found that all LepR-expressing cells in the cerebral cortex were confined to the lower cortical layers, mainly layer 6, contrasting with prior studies that also found LepR-positive cells in upper layers 3 and 4 [25]. Prior studies used a combination of immunohistochemical and in situ hybridization approaches to assess LepR expression in cross-bred reporter mice, while our approach utilized a viral Cre-Lox cell-filling approach in LepR-Cre mice, which enabled a genetic identification of LepR-positive cells and an assessment of their cellular morphology. Non-specific labeling in prior studies may account for some of the observed differences with our findings.

Alternatively, the experimental approach that we employed may have favored cells in lower layers depending on injection depth. However, we consider this unlikely based on 1) the areal extent of labeling and 2) the uniformity of the observed results. On the first issue, labeled cells were found spanning several millimeters in the lower cortical layers. Even if our injections targeted lower layers, it is unlikely that injections would spread only across the lower layers and not vertically into the upper layers (Figure 1, Supplementary Figure S1). Secondly, given the large number of cortical injections (n = 23), it is highly unlikely that none of the injections filled the upper cortical layers. Furthermore, the lower layer distribution corresponds with that observed in LepR-Cre animals crossed with ‘floxed’ fluorescent reporter mice [26]. Therefore, we conclude that the lower layer labeling was likely not an outcome of injection placement. Consequently, based on the specificity and uniformity of these results, we predict that functional LepRs are primarily expressed in the lower cortical layers, particularly layer 6.

We found that the majority of LepR-positive cells in the cortex were neuronal, with a minority being astrocytic [11]. Our viral cell-filling approach enabled an advantageous assessment of the cellular morphology of LepR-positive cells, which lacked pronounced arborizations in the lower cortical layers, suggesting that their effects are confined to local cortical operations. We found that most of these neurons are GABAergic, with a significant fraction being parvalbumin-positive, but not positive for somatostatin or 5-HT3R. Additional assessment is needed to further classify other possible LepR-positive neuronal subtypes.

In addition, we found that astrocytes in the cortex, but not the hippocampus, also express LepR, based on immunolabeling with either GFAP or S100β [27]. LepR in glial cells, including astrocytes, play unique physiological roles [28]. The deletion of astrocytic LepRs in mice results in higher numbers of glial cells. In the hypothalamus, astrocytes contribute to feeding regulation [29]. In addition, mice with astrocytic LepR deletion are more likely to be obese; however, no causal mechanism has been identified in this process [28]. The localization of LepR-positive astrocytes in the same cortical layers as LepR-positive neurons suggests a supportive and possibly synergistic interaction of these cell types.

The localization of LepR-positive neurons and glia in the lower cortical layers suggest that they modulate corticofugal output pathways, which are essential for the top-down processing of information in the brain. In particular, pyramidal neurons in cortical layers 5 and 6 project to the thalamus, which acts as a central orchestrator for information processing, receiving and transmitting signals across vast regions of the cortex [12,14,30,31]. Our findings suggest that the majority of LepR-positive neurons are inhibitory. As such, we speculate that an increase in leptin results in the inhibition of top-down, corticofugal pathways, which could be physiologically related to a reduction in scavenging behavior after feeding. Conversely, decreased firing of the LepR inhibitory neurons, such as in low-leptin states, could facilitate information flow from the cortex to the thalamus; this might contribute to heightened sensory discrimination during periods of active food seeking. Future experiments should seek to characterize the physiological and behavioral roles of cortical LepR neurons.

We also found LepR-expressing neurons in the hippocampus in the CA3 subfield, the GrDG, and the PoDG, confirming prior studies [25]. LepRs have also been reported in the CA2 subfield and the dentate gyrus more generally. The involvement of the hippocampus is of particular interest due to its involvement in learning and memory [32,33,34]. Our present study is limited in the characterization of the specific cell types of LepR-positive neurons, which require further investigation.

Overall, the physiological and behavioral effects of LepRs in the neurons and astrocytes of the mouse forebrain remain to be determined. However, it is known that LepR-expressing neurons in the dorsomedial hypothalamic nucleus (DMH) become depolarized in the presence of leptin, which partly contributes to an increased firing frequency of these neurons [35]. This suggests that leptin binding to its receptor may also excite neurons in the mouse forebrain. Future studies will involve further cell-type-specific dissection of LepR expression in the forebrain. While we were able to characterize a majority of LepR-positive cell types in the cortex, further parcellation is likely necessary, particularly for the hippocampus, since other interneuronal or glial subtypes likely also express LepRs. Finally, future attention should assess the neural circuits in which these cells are embedded and their effects on behavior.

## 4. Materials and Methods

### 4.1. Animals

Adult LepR-Cre mice (Aged: 6–9 mos; Strain: #032457) were obtained from the Jackson Laboratory (Bar Harbor, ME, USA) and the bred offspring of these mice were used in this study. The mice were housed in the vivarium of the LSU School of Veterinary Medicine in a temperature and humidity-controlled room on a 12-h light/dark cycle. Food and water were provided to the mice ad libitum. The Institutional Animal Care and Use Committee (IACUC) of the Louisiana State University approved these protocols.

### 4.2. Stereotactic Surgery and Tracing Experiments

Stereotactic surgeries were performed on male and female mice (n = 29) that had reached a weight of at least 25 g. Animals were first anesthetized via an intraperitoneal injection of a ketamine (100 mg/kg body weight) and xylazine (20 mg/kg body weight) cocktail. Animals were confirmed unconscious when the toe pinch withdrawal reflex was negative. The scalp was then shaved with an electric razor before the head was fixed into a stereotactic instrument (Stoelting, Wood Dale, IL, USA). Eye drops were applied to protect the eyes and 0.1 mL of buprenorphine (0.1 mg/kg) was administered subcutaneously for pre-operative pain management. The scalp was cleaned with iodine and 70% ethanol applied with cotton-tipped applicators. Topical lidocaine was then applied with a separate cotton-tipped applicator before a vertical midline incision was made. The skull was cleaned with 3% hydrogen peroxide, which allowed for greater visualization of the cranial sutures and aided hemostasis. Target regions were localized using the Mouse Brain Atlas as a guide [36]. Craniotomies were performed using a dental micro-drill above the injection site.

A floxed adenovirus vector (UNC Vector Core, Chapel Hill, NC, USA), expressing either EGFP or mCherry, was injected into the brain regions of interest. This cell-type-specific filling approach enabled both the identification of LepR-positive cells and assessment of their morphologies. A 5 µL Neuros syringe with a 33-gauge needle (Hamilton, Reno, NV, USA) was used to inject 1 µL of the viral solution into the target area. The needle was slowly lowered to the targeted area and then left for 3 min to equilibrate with the surrounding tissue. The injection proceeded at a rate of 0.04 µL/min using a micro syringe pump (World Precision Instruments, Sarasota, FL, USA). Bilateral injections were performed with the following coordinates in separate mice. Auditory cortex (n = 7): AP −2.92 mm; ML ± 4.00 mm; DV −2.25 mm; visual cortex (n = 5): AP −2.70 mm; ML ± 2.50 mm; DV −1.00 mm; somatosensory cortex (n = 6): AP −2.06 mm; ML ± 3.00 mm; DV −1.50 mm; insular cortex (n = 5): anterior–posterior (AP) −1.34 mm; medial–lateral (ML) ± 2.75 mm; and dorsal–ventral (DV) −1.75 mm; hippocampus (n = 5): AP −2.54 mm; ML ± 2.50 mm; DV −2.00 mm. Once the injection was completed, the needle remained in place for 3 min to allow equilibration of the mixture before the needle was removed.

Following injections, the surgical area was cleaned, and the scalp was sutured closed. A generic triple antibiotic cream was then applied to the surgical area and an additional 0.1 mL of buprenorphine (0.1 mg/kg) was injected subcutaneously to assist with post-operative pain management. The mouse was then removed from the stereotactic instrument and transferred to a warm cage where it was kept until it awoke from the anesthesia. After awakening, the animal was then transferred to its normal cage, where it was kept for 21 days until sacrifice.

### 4.3. Histology

To characterize the expression of the viral encoded fluorophores, the mice were sacrificed following anesthesia with isoflurane until a toe pinch failed to elicit the withdrawal reflex. Each mouse was then transcardially perfused with 20 mL of PBS solution (10 mM, pH of 7.4) and then slowly perfused with 10 mL of a 4% PFA solution. Each mouse was decapitated and the whole brain was removed and placed in the 4% PFA solution for 48 h at 4 °C. The brains were then transferred to a 4% PFA/30% sucrose solution at 4 °C for cryopreservation. The brains were coronally sectioned at a thickness of 50 µm using a freezing microtome (Leica Biosystems, Deer Park, IL). The sections were then transferred to a 48-well plate containing PBS until staining.

Regions with the injected AAV were then immunostained for cell-type-specific markers of neurons and astrocytes. For all stains, the sections were transferred to a separate well plate containing excess PBS for washing. After three successive five-minute washes, the sections were transferred to a well containing 500 µL of a blocking solution (PBS with normal goat serum) for one hour. The sections were then transferred to a well containing a 1:1000 dilution in PBS of the primary antibodies NeuN (ab104225, Abcam, Cambridge, UK), S100β (AMAB91038, Sigma-Aldrich, St. Louis, MO, USA), GFAP (16825-1-AP, Proteintech, Rosemont, IL, USA), VGlut2 (ab21643, Abcam, Cambridge, UK), GAD67 (ab228710, Abcam, Cambridge, UK), parvalbumin (ab11427, Abcam, Cambridge, UK), somatostatin (sc74456, Santa Cruz Biotechnology, Dallas, TX, USA), and serotonin type 3 receptor (pc347, Sigma-Aldrich, St. Louis, MO, USA). The incubations took place overnight, in the dark, at 4 °C. The following day, the sections were again thrice washed in PBS before being transferred to the secondary antibody, goat-anti-rabbit and goat-anti-mouse, as appropriate, and allowed to incubate for an hour. Secondary antibodies were used that were pre-conjugated with an AlexaFluor tag (Abcam, Cambridge, UK) and diluted at a 1:1000 ratio.

For the staining of perineuronal nets (PNNs), sections were first incubated for 15 min in a blocking solution containing avidin and biotin solutions (#SP-2002, Vector Laboratories, Burlingame, CA, USA). Next, sections were thrice rinsed in PBS and then incubated with a 1:500 dilution of biotinylated WFA/WFL (Wisteria floribunda agglutinin/lectin) (#B-1355, Vector Laboratories, Burlingame, CA, USA) overnight at room temperature. The following day, sections were thrice rinsed in PBS before incubation in a 1:500 diluted solution of streptavidin conjugated with Alexa Fluor 568 (CF-29035, Biotium, CA, USA) for 1 h at room temperature.

The histologically processed sections were mounted on gelatin-coated slides and then cover-slipped using Vectashield antifade mounting media with DAPI (#H-1500, Vector Laboratories, Burlingame, CA, USA). The slides were stored at 4 °C until imaged.

### 4.4. Imaging and Quantification

Fluorescent images were obtained using a Nanozoomer slide scanner (Hamamatsu Photonics, Bridgewater, NJ, USA) with semi-automatic settings. The whole-slide image data were then viewed and used to generate regions of interest. Regions of interest were imaged further with a confocal Zeiss microscope (Oberkochen, Baden-Württemberg, Germany). Acquired images were then analyzed using Fiji software (Version 2.14.0, NIH, Bethesda, MD, USA). Sholl analyses were conducted using the Neuroanatomy SNP plugin in Fiji (Version 2.14.0, NIH). Single- and double-labeled cells were counted and averaged from three sections in each experiment. Counts were averaged and reported as a percentage of LepR-positive cells. Data were statistically assessed using Prism (Version 10.3.0, Graphpad, San Diego, CA, USA).

## Data Availability

The original data presented in the study are openly available from Zenodo at DOI: https://doi.org/10.5281/zenodo.13137435.

## Supplementary Materials

The following supporting information can be downloaded at: https://www.mdpi.com/article/10.3390/ijms25189854/s1.
