# Supplementary material for: Cell-Type-Specific Expression of Leptin Receptors in the Mouse Forebrain
Source: Int J Mol Sci. 2024 Sep 12;25(18):9854. doi: 10.3390/ijms25189854 (PMC11432612; doi:10.3390/ijms25189854)
Supplement: Supplementary file 1 [file ijms-25-09854-s001.zip › ijms-3161498-supplementary.pdf]

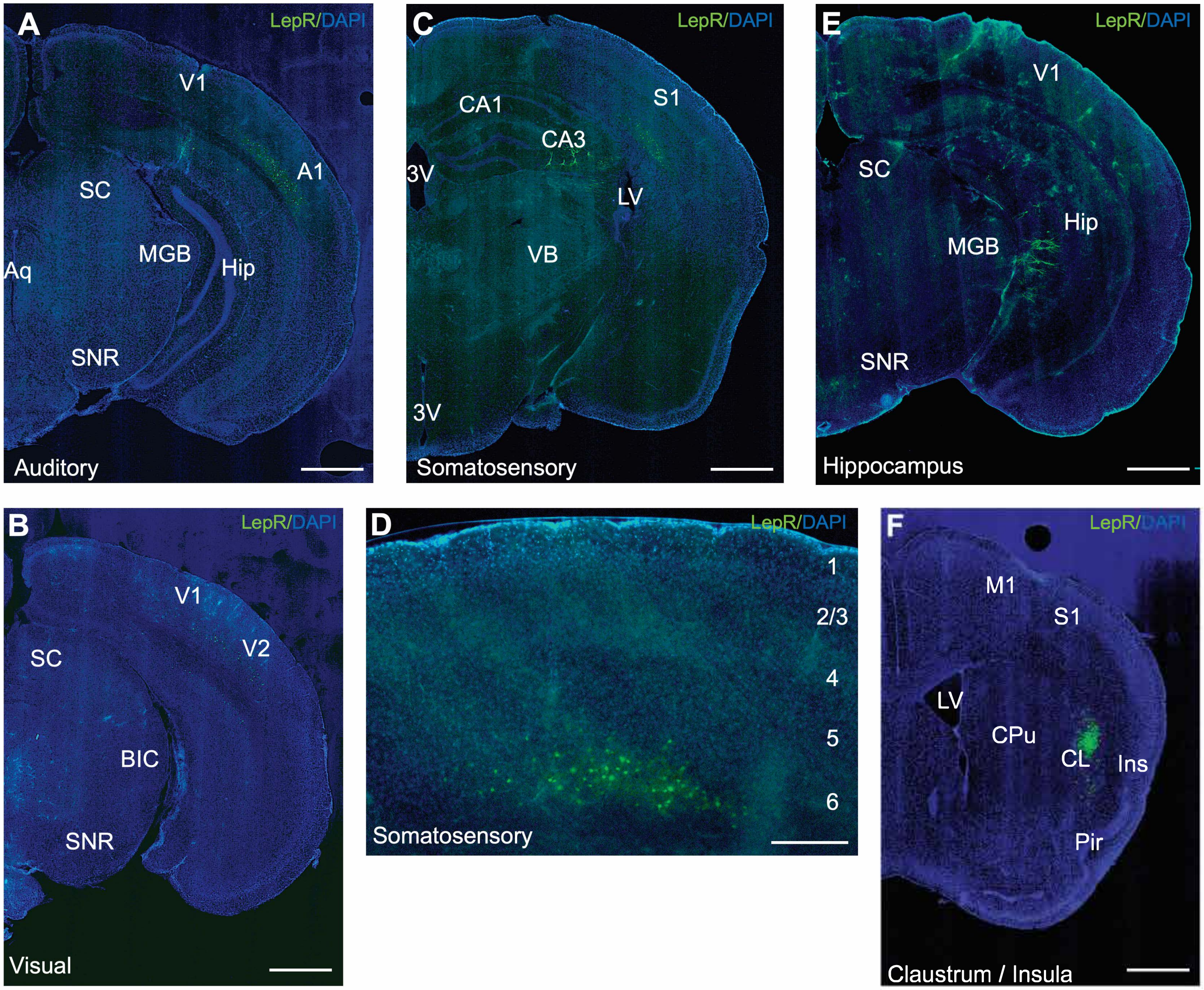

Supplementary Figure S1. Low-magnification images of LepR-positive neurons (green) with DAPI counterstain (blue) (**A-B**, **E-F**) corresponding to high magnification images in Figs.1 and 5. Scale bars: 1 mm. Low- (**C**) and high- (**D**) magnification images of somatosensory cortical LepR-positive neurons (green) with DAPI (blue). Scale bar in *C*: 1mm. Scale bar in *D*: 200 μm

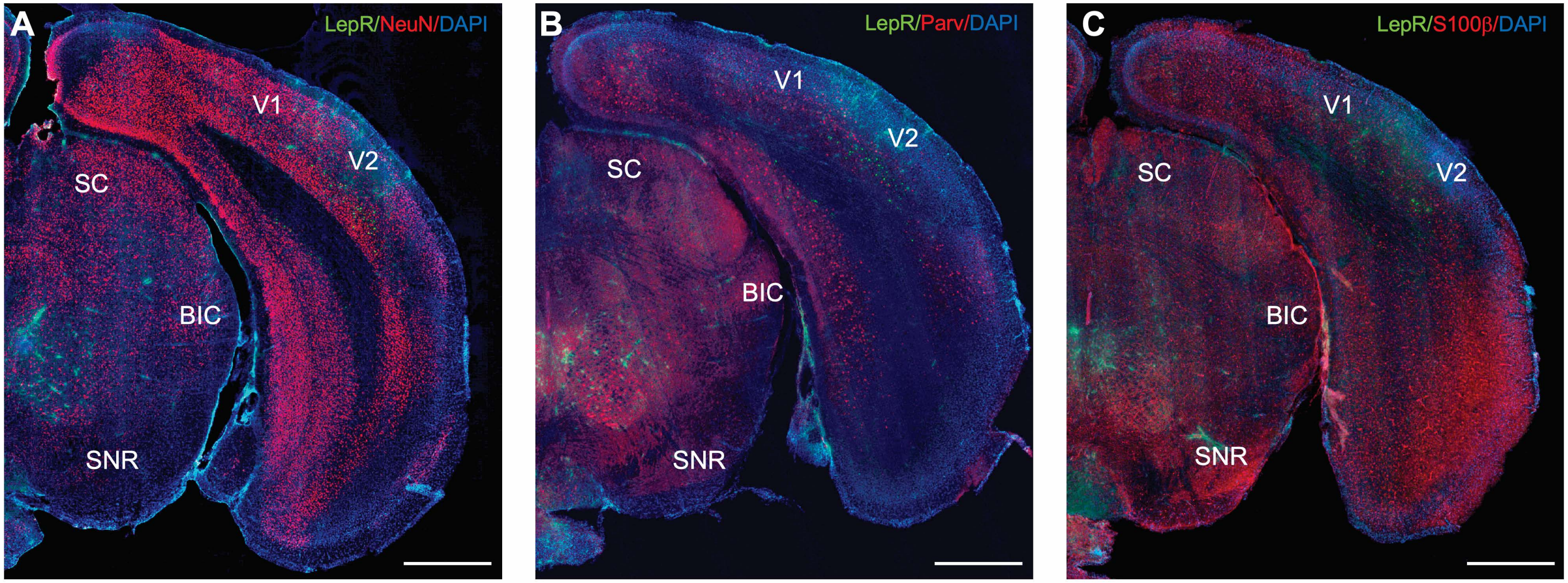

Supplementary Figure S2. Low-magnification images of LepR-positive neurons (green) in the visual cortex co-immunostained for neurochemical markers (red). (**A**) Neuronal marker NeuN, (**B**) parvalbumin, (**C**) S100β. DAPI counterstain in blue. Images are from the same respective sections as the high-magnification images in Fig.2. Scale bar: 1mm. BIC: brachium of the inferior colliculus, SC: superior colliculus, SNR: substantia nigra reticular part, V1: primary visual cortex, V2, secondary visual cortex

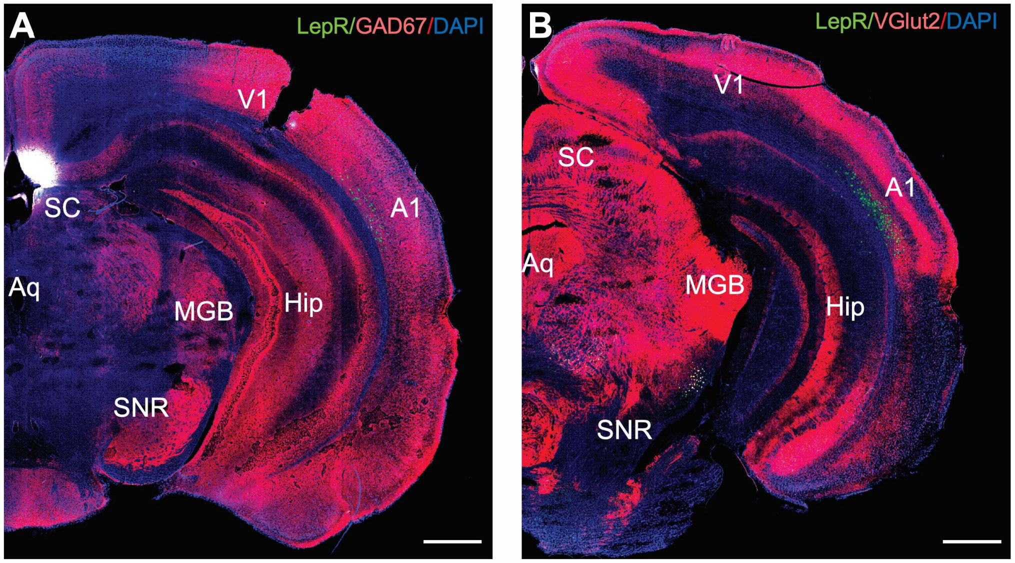

Supplementary Figure S3. Low-magnification images of LepR-positive neurons (green) in the visual cortex co-immunostained for GAD67 (**A**) or VGlut2 (**B**). DAPI counterstain is in blue. Images are from the same respective sections as the high-magnification images in Fig.3. Scale bar: 1mm. Aq: midbrain aqueduct, BIC: brachium of the inferior colliculus, Hip: hippocampus, SC: superior colliculus, SNR: substantia nigra reticular part, V1: primary visual cortex, V2, secondary visual cortex

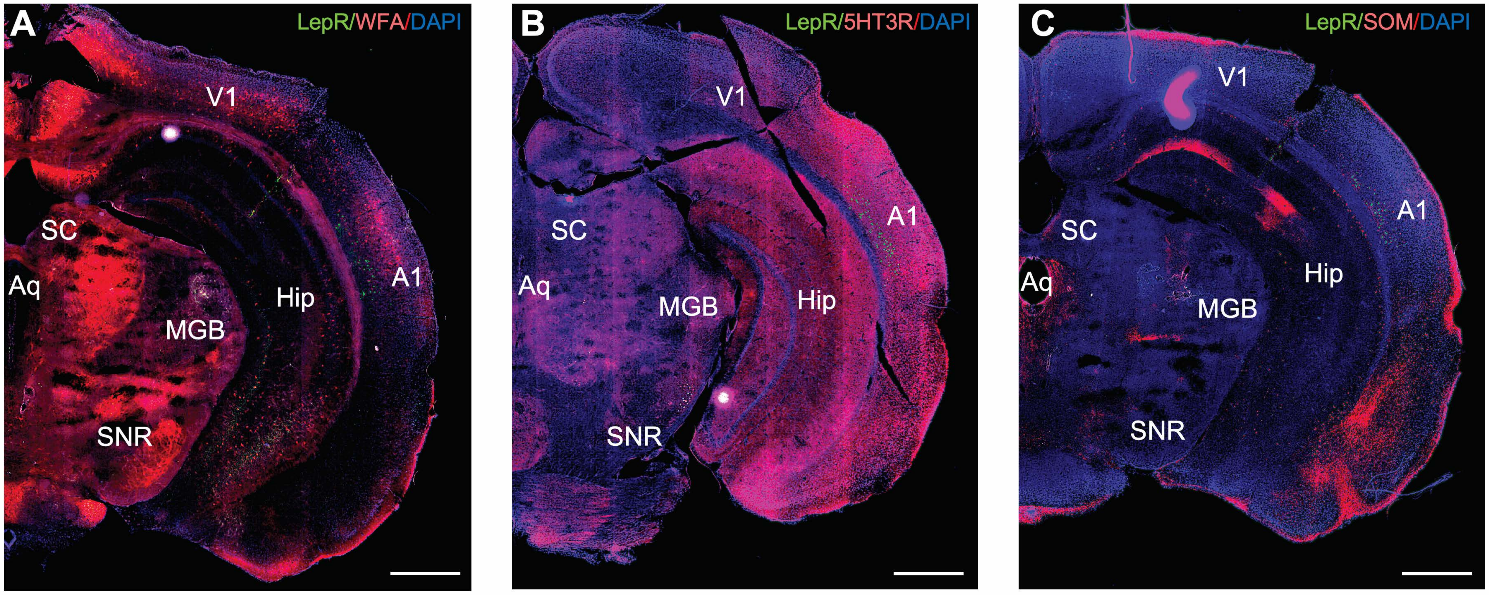

Supplementary Figure S4. Low-magnification images of LepR-positive neurons (green) in the visual cortex co-labeled for perineuronal nets (**A**, WFA), the serotonin type 3 receptor (**B**), or somatostatin (**C**). DAPI counterstain is in blue. Images are from the same respective sections as the high-magnification images in Fig. 4. Scale bar: 1mm. Aq: midbrain aqueduct, BIC: brachium of the inferior colliculus, Hip: hippocampus, SC: superior colliculus, SNR: substantia nigra reticular part, V1: primary visual cortex, V2, secondary visual cortex

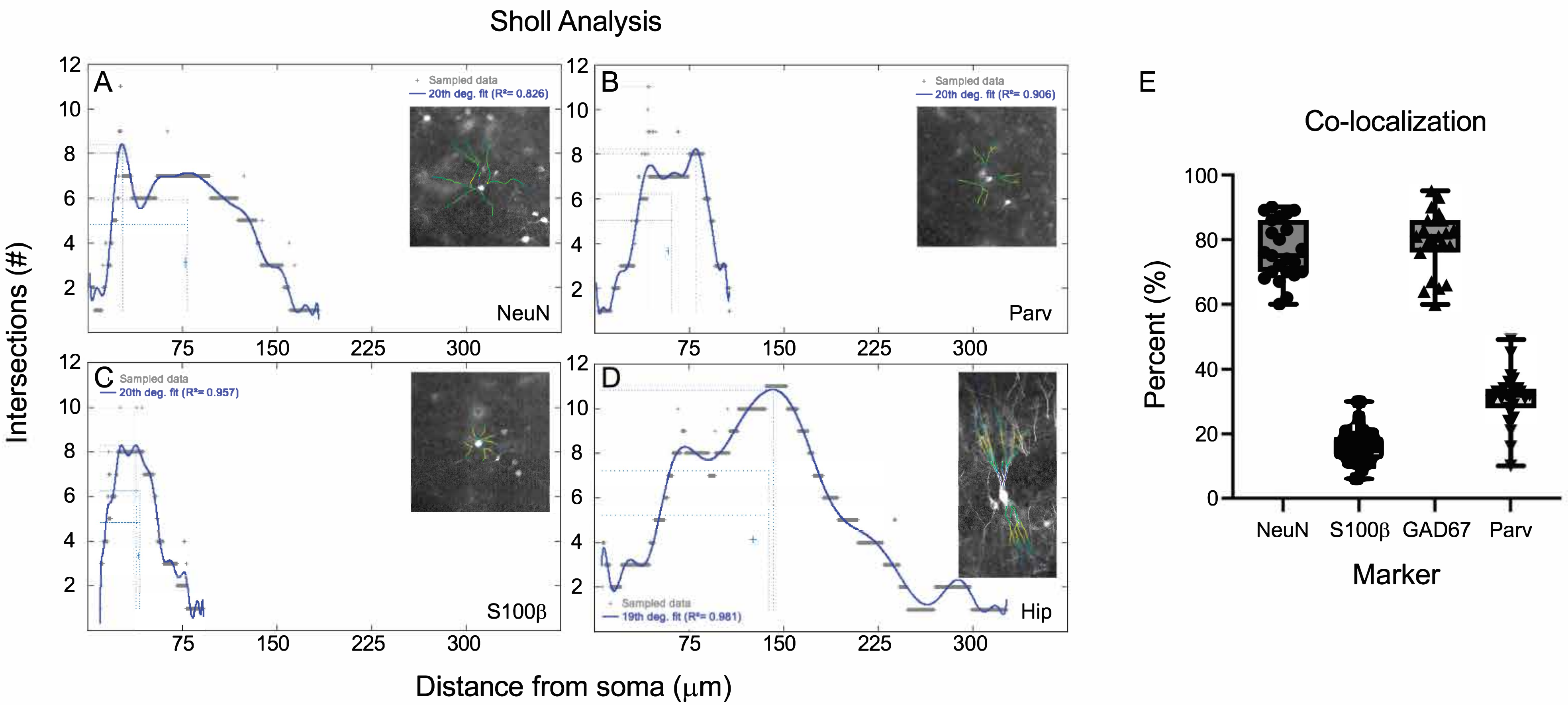

Supplementary Figure S5. Sholl analysis and co-localization of LepR-positive neurons in the cerebral cortex and hippocampus. Linear plots for the Sholl analysis of arborization of representative LepR-positive neurons that are co-labeled for NeuN (**A**), parvalbumin (**B**), S100β (**C**), and in the cerebral cortex and for NeuN in the hippocampus (**D**). Grey points in each plot show sampled data and the blue line depicts the best curve fit to the data. Inset images show representative analyzed cells from Fig. 2, with colored nodes depicted on traced dendrites. Box plot with points (**E**) summarizing the percent distribution of LepR-positive neurons that were double labeled for NeuN, S100β, GAD67, and parvalbumin in the cortex.
